# Supplementary material for: Unusual outcome variances as a method to identify potentially problematic clinical trials
Source: PLoS One. 2026 Apr 15;21(4):e0346238. doi: 10.1371/journal.pone.0346238 (PMC13082665; doi:10.1371/journal.pone.0346238)
Supplement: S1 Text — (DOCX) [file pone.0346238.s001.docx]

S1 Text: Supplementary Materials for “Unusual Outcome Variances as a Method to Identify Potentially Problematic Clinical Trials**”**

*Two DiVBTA ratio measures*

For notation, let s_T_, x̄_T_, and n_T_ represent the standard deviation, mean, and sample size respectively in the treatment group, and let s_C_, x̄_C_ and n_C_ represent the standard deviation, mean, and sample size respectively in the control group.

*a) Assessing statistical significance for three DiVBTA measures for parallel arm trials:*

- The ratio of variances ($s_{T}^{2}/s_{c}^{2}$) follows an F distribution and the standard error of the ratio of variances under the null is ($\sqrt{2{df}_{C}^{2}({df}_{T}+{df}_{C}-2)/({df}_{T}\left( {df}_{C}-2 \right)^{2}\left( {df}_{C}-4 \right))}$) where df_C_  and df_T_ stands for degrees of freedom in the control and treatment group respectively. An F test with n_T_ − 1, n_C_ − 1 degrees of freedom is used to test whether the ratio of variances is significantly different from 1 under the null hypothesis.

- The log of the ratio of SDs, frequently referred to as LnVR in the literature ($ln\left( \frac{S_{T}}{S_{C}} \right)+ \frac{1}{2\left( N_{T}-1 \right)}-\frac{1}{2(N_{C}-1)} )$ and the standard error of the lnVR ($\sqrt{\frac{1}{2\left( n_{T}-1 \right)}+\frac{1}{2\left( n_{C}-1 \right)}})$ has an approximate normal distribution. The significance under the null hypothesis can be approximated the degrees of freedom based on the Welch–Satterthwaite approximation.
