# Supplementary material for: Unusual outcome variances as a method to identify potentially problematic clinical trials
Source: PLoS One. 2026 Apr 15;21(4):e0346238. doi: 10.1371/journal.pone.0346238 (PMC13082665; doi:10.1371/journal.pone.0346238)
Supplement: S1 Table — (DOCX) [file pone.0346238.s002.docx]

S1 Table. Specificity of 4-sigma statistically-significant lnCVR when randomization and heterogeneous treatment effects occur in legitimate trials, by sample size per trial arm.

| **HbA1c Effect size** | **Subgroup with HTE 10%** | | **Subgroup with HTE 20%** | | **Subgroup with HTE 30%*** | | **Subgroup with HTE 40%**^*^ | | **Subgroup with HTE 50%**^*^ | |
| --- | --- | --- | --- | --- | --- | --- | --- | --- | --- | --- |
|  | n=20 | n=250 | n=20 | n=250 | n=20 | n=250 | n=20 | n=250 | n=20 | n=250 |
| -0.0% | 97.2% | 99.9% | 98.5% | 100.0% | 98.2% | 100.0% | 98.6% | 100.0% | 98.1% | 100.0% |
| -0.2% | 97.1% | 100.0% | 97.8% | 100.0% | 98.0% | 100.0% | 98.0% | 100.0% | 98.0% | 100.0% |
| -0.4% | 97.4% | 100.0% | 96.9% | 100.0% | 97.0% | 100.0% | 98.3% | 100.0% | 97.1% | 100.0% |
| -0.6% | 98.2% | 100.0% | 97.6% | 100.0% | 97.9% | 100.0% | 97.3% | 100.0% | 98.0% | 100.0% |
| -0.8% | 97.3% | 100.0% | 97.4% | 100.0% | 97.7% | 100.0% | 97.1% | 99.9% | 97.1% | 100.0% |
| -1.0% | 98.1% | 100.0% | 97.2% | 99.9% | 96.5% | 100.0% | 95.9% | 100.0% | 97.3% | 99.8% |
| -1.2% | 98.0% | 100.0% | 96.7% | 100.0% | 95.9% | 99.8% | 95.3% | 100.0% | 94.5% | 99.9% |
| -1.4%^*^ | 97.7% | 100.0% | 96.8% | 99.9% | 95.0% | 99.9% | 93.8% | 99.6% | 93.6% | 99.7% |
| -1.6%^*^ | 96.8% | 99.9% | 95.3% | 100.0% | 93.3% | 99.6% | 91.9% | 99.8% | 90.2% | 98.8% |
| -1.8%^*^ | 96.7% | 100.0% | 95.0% | 99.7% | 91.9% | 99.5% | 87.5% | 99.1% | 87.0% | 98.2% |
| -2.0%^*^ | 96.6% | 99.8% | 94.0% | 99.9% | 87.7% | 99.3% | 82.3% | 98.3% | 80.1% | 96.6% |

^*^ The systematic review of diabetes trials included in this report suggests that treatment effects larger than a -1.2% improvement and subgroup effects larger than 30% may not reflect standard trial dynamics. Specifically, the 3-sigma bounds of treatment effect sizes ranged from -1.4% to +0.8% and the meta-analysis of lnCVRs did not find convincing evidence of a statistically significant LnCVR summary statistics, suggesting that trial where large subgroups (e.g., 40%) respond differently in the intervention arm are unlikely.
