# Supplementary material for: Unusual outcome variances as a method to identify potentially problematic clinical trials
Source: PLoS One. 2026 Apr 15;21(4):e0346238. doi: 10.1371/journal.pone.0346238 (PMC13082665; doi:10.1371/journal.pone.0346238)
Supplement: S2 Table — (DOCX) [file pone.0346238.s003.docx]

S2 Table. Specificity of 4-sigma statistically significant lnCVR when randomization and an extreme form MNAR dropout (0–50%) occur in trials, by sample size per trial arm.

| Prevalence of missing-not-at-random dropout level | Sample size per clinical trial arm | |
| --- | --- | --- |
|  | n=20 | n=250 |
| 0 | 97.7% | 100.0% |
| 10% | 95.2% | 99.7% |
| 20% | 90.9% | 98.2% |
| 30%^*^ | 84.8% | 95.4% |
| 40%^*^ | 77.3% | 88.3% |
| 50%^*^ | 69.0% | 82.5% |

^*^ 30% and larger dropout rates are typically not considered standard trial dynamics.
