# Supplementary material for: Unusual outcome variances as a method to identify potentially problematic clinical trials
Source: PLoS One. 2026 Apr 15;21(4):e0346238. doi: 10.1371/journal.pone.0346238 (PMC13082665; doi:10.1371/journal.pone.0346238)
Supplement: S3 Table — (DOCX) [file pone.0346238.s004.docx]

S3 Table. Sensitivity of 4-sigma statistically significant lnCVR for detecting simulated fraud (50–90% worst HbA1c scores in intervention arm replaced by best-responder value), by sample size per trial arm.

| Proportion ‘worst’ responders deleted and replaced by “best” response. | Sample size per clinical trial arm | |  |
| --- | --- | --- | --- |
|  | n=20 | n=250 |  |
| 50%^*^ | 21.9% | 2.7% |  |
| 60%^*^ | 34.6% | 2.4% |  |
| 70%* | 59.0% | 2.8% |  |
| 80%^*^ | 83.5% | 17.1% |  |
| 90%^*^ | 99.0% | 73.8% |  |

^*^ Deleting 50% or more of the individual participant data cannot be considered subtle or minimal data fabrication. Stated differently, these simulation statistics evaluate the sensitivity to detect massive data fraud.
