# Supplementary figures and images for: Unusual outcome variances as a method to identify potentially problematic clinical trials
Source: PLoS One. 2026 Apr 15;21(4):e0346238. doi: 10.1371/journal.pone.0346238 (PMC13082665; doi:10.1371/journal.pone.0346238)

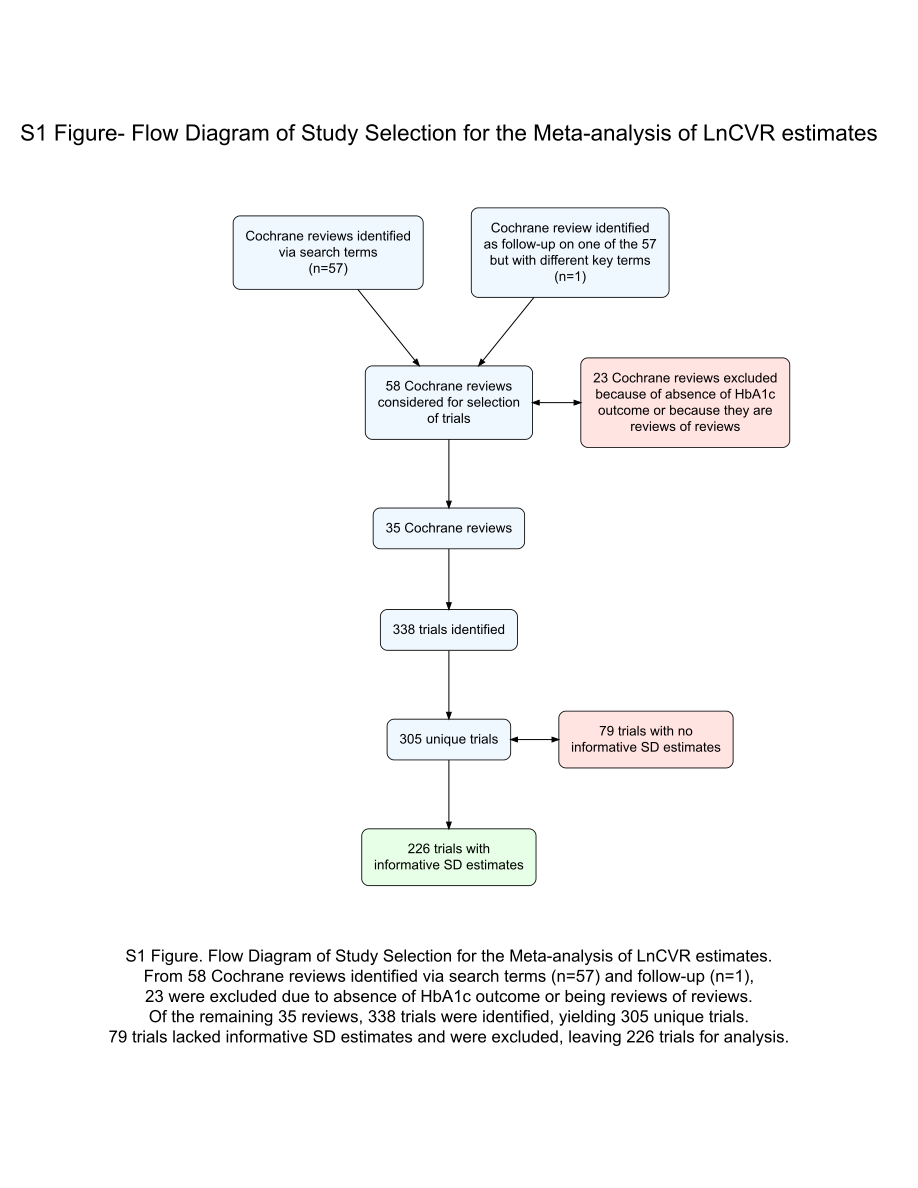

Supplement: S1 Fig — From 58 Cochrane reviews identified via search terms (n = 57) and follow-up (n = 1), 23 were excluded due to absence of HbA1c outcome or being reviews of reviews. 338 trials were identified in the remaining 35 Cochrane reviews, yielding 305 unique trials. 79 trials lacked informative SD estimates and were excluded, leaving 226 trials for the lnCVR meta-analysis. (PNG) [file pone.0346238.s005.png]
