# Supplementary material for: Unusual outcome variances as a method to identify potentially problematic clinical trials
Source: PLoS One. 2026 Apr 15;21(4):e0346238. doi: 10.1371/journal.pone.0346238 (PMC13082665; doi:10.1371/journal.pone.0346238)
Supplement: S4 Table — (DOCX) [file pone.0346238.s006.docx]

**S4 Table - Characteristics of trials by presence or absence of post-randomization standard deviations**

| **Characteristic** | No standard deviation information  N = 79^1^ | Standard deviation information available  N = 226^1^ | **p-value**^2^ |
| --- | --- | --- | --- |
| **Reported funding source** |  |  | <0.001 |
| both | 6.0 (7.6%) | 24.0 (10.6%) |  |
| commercial | 50.0 (63.3%) | 61.0 (27.0%) |  |
| non-commercial | 16.0 (20.3%) | 89.0 (39.4%) |  |
| not reported | 7.0 (8.9%) | 52.0 (23.0%) |  |
| **Indexed in Pubmed** |  |  | 0.013 |
| Not PubMed Indexed | 2.0 (2.5%) | 27.0 (11.9%) |  |
| PubMed Indexed | 77.0 (97.5%) | 199.0 (88.1%) |  |
| **Country study was conducted in** |  |  | <0.001 |
| Countries with high retraction rate | 0.0 (0.0%) | 38.0 (16.8%) |  |
| Other countries | 79.0 (100.0%) | 188.0 (83.2%) |  |
| **Unpublished data sources** |  |  | 0.405 |
| published and unpublished data | 12.0 (15.2%) | 46.0 (20.4%) |  |
| published data only | 67.0 (84.8%) | 180.0 (79.6%) |  |
| **Cross-over trials** |  |  | 0.148 |
| cross-over | 3.0 (3.8%) | 21.0 (9.3%) |  |
| parallel | 76.0 (96.2%) | 205.0 (90.7%) |  |
| **Quality score for randomization** |  |  | 0.001 |
| Mean (SD) | 1.6 (0.5) | 1.4 (0.5) |  |
| Min - Max | 0.5 - 2.0 | 0.0 - 2.0 |  |
| Median (Q1, Q3) | 2.0 (1.0, 2.0) | 1.3 (1.0, 2.0) |  |
| **Quality score of blinding** |  |  | 0.285 |
| Mean (SD) | 1.1 (0.7) | 1.0 (0.7) |  |
| Min - Max | 0.0 - 2.0 | 0.0 - 2.0 |  |
| Median (Q1, Q3) | 1.0 (0.5, 2.0) | 1.0 (0.3, 1.5) |  |
| (Missing) | 1 | 6 |  |
| **Quality score for ascertainment** |  |  | 0.455 |
| Mean (SD) | 1.5 (0.7) | 1.3 (0.8) |  |
| Min - Max | 0.0 - 2.0 | 0.0 - 2.0 |  |
| Median (Q1, Q3) | 2.0 (1.0, 2.0) | 2.0 (1.0, 2.0) |  |
| (Missing) | 1 | 6 |  |
| **Max HbA1c effect size** |  |  | 0.957 |
| Mean (SD) | -0.4 (0.5) | -0.4 (0.6) |  |
| Min - Max | -2.0 - 0.7 | -2.9 - 1.6 |  |
| Median (Q1, Q3) | -0.3 (-0.7, -0.1) | -0.3 (-0.7, -0.1) |  |
| **Sample size** |  |  | <0.001 |
| Mean (SD) | 430.7 (560.2) | 190.7 (775.7) |  |
| Min - Max | 13.0 - 4351.0 | 5.0 - 11140.0 |  |
| Median (Q1, Q3) | 310.0 (120.0, 533.0) | 65.5 (36.0, 157.0) |  |
| **Number of trial arms** |  |  | 0.007 |
| Mean (SD) | 2.6 (1.4) | 2.2 (0.6) |  |
| Min - Max | 2.0 - 10.0 | 2.0 - 6.0 |  |
| Median (Q1, Q3) | 2.0 (2.0, 3.0) | 2.0 (2.0, 2.0) |  |
| **Duration of trial** |  |  | 0.005 |
| Mean (SD) | 9.0 (9.4) | 7.3 (7.6) |  |
| Min - Max | 2.0 - 60.0 | 0.0 - 60.0 |  |
| Median (Q1, Q3) | 6.0 (6.0, 12.0) | 6.0 (3.0, 6.0) |  |
| (Missing) | 1 | 1 |  |
| **Number of authors** |  |  | 0.037 |
| Mean (SD) | 6.8 (2.8) | 6.1 (3.4) |  |
| Min - Max | 3.0 - 18.0 | 1.0 - 23.0 |  |
| Median (Q1, Q3) | 6.0 (5.0, 9.0) | 6.0 (4.0, 8.0) |  |
| (Missing) | 2 | 3 |  |
| **Publication year** |  |  | 0.857 |
| Mean (SD) | 2007.0 (5.2) | 2006.2 (8.2) |  |
| Min - Max | 1989.0 - 2021.0 | 1982.0 - 2021.0 |  |
| Median (Q1, Q3) | 2008.0 (2003.0, 2010.0) | 2008.0 (2003.0, 2011.0) |  |
| **Data available to calculate Carlisle-Fisher-Stouffer p-value** |  |  | >0.999 |
| Available | 74.0 (93.7%) | 209.0 (92.5%) |  |
| Unavailable | 5.0 (6.3%) | 17.0 (7.5%) |  |
| **Baseline data unbalanced or too balanced (p<0.001)** |  |  | 0.766 |
| p<0.001 | 3.0 (4.1%) | 12.0 (5.7%) |  |
| p>0.001 | 71.0 (95.9%) | 197.0 (94.3%) |  |
| (Missing) | 5 | 17 |  |
| **Baseline data unbalanced or too balanced (p<0.01)** |  |  | 0.805 |
| p<0.01 | 5.0 (6.8%) | 18.0 (8.6%) |  |
| p>0.01 | 69.0 (93.2%) | 191.0 (91.4%) |  |
| (Missing) | 5 | 17 |  |
| **Baseline data unbalanced or too balanced (p<0.05)** |  |  | 0.456 |
| p<0.05 | 9.0 (12.2%) | 34.0 (16.3%) |  |
| p>0.05 | 65.0 (87.8%) | 175.0 (83.7%) |  |
| (Missing) | 5 | 17 |  |
| ^1^n (%) | | | |
| ^2^Fisher's exact test; Wilcoxon rank sum test | | | |
