# Supplementary material for: Unusual outcome variances as a method to identify potentially problematic clinical trials
Source: PLoS One. 2026 Apr 15;21(4):e0346238. doi: 10.1371/journal.pone.0346238 (PMC13082665; doi:10.1371/journal.pone.0346238)
Supplement: S5 Table — (DOCX) [file pone.0346238.s007.docx]

**S5 Table - Characteristics of trials by with and without problematic lnCVR**

| **Characteristic** | **Non-problematic lnCVR**  N = 207^1^ | **Problematic lnCVR**  N = 19^1^ | **p-value**^2^ |
| --- | --- | --- | --- |
| **Reported funding source** |  |  | 0.44 |
| both | 23.0 (11.1%) | 1.0 (5.3%) |  |
| commercial | 55.0 (26.6%) | 6.0 (31.6%) |  |
| non-commercial | 79.0 (38.2%) | 10.0 (52.6%) |  |
| not reported | 50.0 (24.2%) | 2.0 (10.5%) |  |
| **Indexed in Pubmed** |  |  | >0.99 |
| Not PubMed Indexed | 25.0 (12.1%) | 2.0 (10.5%) |  |
| PubMed Indexed | 182.0 (87.9%) | 17.0 (89.5%) |  |
| **Country study was conducted in** |  |  | 0.54 |
| Countries with high retraction rate | 34.0 (16.4%) | 4.0 (21.1%) |  |
| Other countries | 173.0 (83.6%) | 15.0 (78.9%) |  |
| **Unpublished data sources** |  |  | 0.77 |
| published and unpublished data | 43.0 (20.8%) | 3.0 (15.8%) |  |
| published data only | 164.0 (79.2%) | 16.0 (84.2%) |  |
| **Cross-over trials** |  |  | 0.69 |
| cross-over | 19.0 (9.2%) | 2.0 (10.5%) |  |
| parallel | 188.0 (90.8%) | 17.0 (89.5%) |  |
| **Quality score for randomization** |  |  | 0.16 |
| Mean (SD) | 1.4 (0.5) | 1.2 (0.4) |  |
| Min - Max | 0.0 - 2.0 | 1.0 - 2.0 |  |
| Median (Q1, Q3) | 1.5 (1.0, 2.0) | 1.0 (1.0, 1.5) |  |
| **Quality score of blinding** |  |  | 0.69 |
| Mean (SD) | 1.0 (0.7) | 0.9 (0.8) |  |
| Min - Max | 0.0 - 2.0 | 0.0 - 2.0 |  |
| Median (Q1, Q3) | 1.0 (0.3, 1.5) | 0.5 (0.3, 2.0) |  |
| (Missing) | 6 | 0 |  |
| **Quality score for ascertainment** |  |  | >0.99 |
| Mean (SD) | 1.3 (0.8) | 1.4 (0.7) |  |
| Min - Max | 0.0 - 2.0 | 0.0 - 2.0 |  |
| Median (Q1, Q3) | 2.0 (1.0, 2.0) | 1.2 (1.0, 2.0) |  |
| (Missing) | 6 | 0 |  |
| **Max HbA1c effect size** |  |  | 0.28 |
| Mean (SD) | -0.4 (0.6) | -0.5 (0.6) |  |
| Min - Max | -2.9 - 1.6 | -2.0 - 0.2 |  |
| Median (Q1, Q3) | -0.3 (-0.6, 0.0) | -0.4 (-0.8, -0.1) |  |
| **Sample size** |  |  | 0.023 |
| Mean (SD) | 202.8 (809.5) | 59.5 (47.2) |  |
| Min - Max | 5.0 - 11140.0 | 16.0 - 195.0 |  |
| Median (Q1, Q3) | 71.0 (37.0, 169.0) | 44.0 (25.0, 66.0) |  |
| **Number of trial arms** |  |  | 0.60 |
| Mean (SD) | 2.2 (0.6) | 2.1 (0.3) |  |
| Min - Max | 2.0 - 6.0 | 2.0 - 3.0 |  |
| Median (Q1, Q3) | 2.0 (2.0, 2.0) | 2.0 (2.0, 2.0) |  |
| **Duration of trial** |  |  | 0.058 |
| Mean (SD) | 7.5 (7.9) | 4.8 (3.2) |  |
| Min - Max | 1.0 - 60.0 | 0.0 - 12.0 |  |
| Median (Q1, Q3) | 6.0 (3.0, 8.0) | 3.0 (3.0, 6.0) |  |
| (Missing) | 1 | 0 |  |
| **Number of authors** |  |  | 0.68 |
| Mean (SD) | 6.2 (3.5) | 5.6 (2.3) |  |
| Min - Max | 1.0 - 23.0 | 2.0 - 9.0 |  |
| Median (Q1, Q3) | 6.0 (4.0, 8.0) | 5.0 (4.0, 7.0) |  |
| (Missing) | 3 | 0 |  |
| **Publication year** |  |  | 0.75 |
| Mean (SD) | 2006.3 (8.2) | 2005.5 (8.7) |  |
| Min - Max | 1982.0 - 2021.0 | 1982.0 - 2015.0 |  |
| Median (Q1, Q3) | 2008.0 (2003.0, 2011.0) | 2007.0 (2003.0, 2011.0) |  |
| **Data available to calculate Carlisle-Fisher-Stouffer p-value** |  |  | 0.64 |
| Available | 192.0 (92.8%) | 17.0 (89.5%) |  |
| Unavailable | 15.0 (7.2%) | 2.0 (10.5%) |  |
| **Baseline data unbalanced or too balanced (p<0.001)** |  |  | 0.062 |
| p<0.001 | 9.0 (4.7%) | 3.0 (17.6%) |  |
| p>0.001 | 183.0 (95.3%) | 14.0 (82.4%) |  |
| (Missing) | 15 | 2 |  |
| **Baseline data unbalanced or too balanced (p<0.01)** |  |  | 0.045 |
| p<0.01 | 14.0 (7.3%) | 4.0 (23.5%) |  |
| p>0.01 | 178.0 (92.7%) | 13.0 (76.5%) |  |
| (Missing) | 15 | 2 |  |
| **Baseline data unbalanced or too balanced (p<0.05)** |  |  | 0.16 |
| p<0.05 | 29.0 (15.1%) | 5.0 (29.4%) |  |
| p>0.05 | 163.0 (84.9%) | 12.0 (70.6%) |  |
| (Missing) | 15 | 2 |  |
| ^1^n (%) | | | |
| ^2^Fisher's exact test; Wilcoxon rank sum test | | | |
