# Supplementary material for: Unusual outcome variances as a method to identify potentially problematic clinical trials
Source: PLoS One. 2026 Apr 15;21(4):e0346238. doi: 10.1371/journal.pone.0346238 (PMC13082665; doi:10.1371/journal.pone.0346238)
Supplement: S6 Table — Bootstrap summary of lnCVR effect estimates and 3σ prediction interval bounds. Results from 1000 bootstrap replications (with replacement at the study level). The original values are based on the full dataset (n = 226 studies). The 95% confidence intervals are percentile-based. (DOCX) [file pone.0346238.s008.docx]

**S6-Table:Bootstrap sampling to assess robustness of 3-sigma bounds towards trial selection**

| **Variable** | **Original** | **Bootstrap Mean** | **Bootstrap SE** | **95% CI** |
| --- | --- | --- | --- | --- |
| lower_lncvr3sigma | -0.578 | -0.561 | 0.053 | (-0.67, -0.46) |
| upper_lncvr3sigma | 0.534 | 0.517 | 0.065 | (0.388, 0.65) |
| X_Intercept | -0.022 | -0.022 | 0.017 | (-0.054, 0.011) |
| median_LnCVR | -0.024 | -0.029 | 0.016 | (-0.064, -0.003) |
| fenceLnCVR_up.75% | 0.497 | 0.515 | 0.059 | (0.414, 0.63) |
| fenceLnCVR_low.25% | -0.567 | -0.586 | 0.051 | (-0.71, -0.499) |
