# Supplementary material for: Unusual outcome variances as a method to identify potentially problematic clinical trials
Source: PLoS One. 2026 Apr 15;21(4):e0346238. doi: 10.1371/journal.pone.0346238 (PMC13082665; doi:10.1371/journal.pone.0346238)
Supplement: Attachment — Submitted filename: Response to reviewers.docx [file pone.0346238.s010.docx]

**Comments to the Author**

**1. Is the manuscript technically sound, and do the data support the conclusions?

The manuscript must describe a technically sound piece of scientific research with data that supports the conclusions. Experiments must have been conducted rigorously, with appropriate controls, replication, and sample sizes. The conclusions must be drawn appropriately based on the data presented.**

**Reviewer #1: Partly**

**Reviewer #2: Yes**

Extensive revisions and additions are presented addressing Reviewer #1’s (Dr. Richter’s) comments.

**2. Has the statistical analysis been performed appropriately and rigorously?**

**Reviewer #1: No**

**Reviewer #2: Yes**

The information and background work on the statistical methodology has been expanded and extensive simulation work has been added.

**3. Have the authors made all data underlying the findings in their manuscript fully available?

The** [**PLOS Data policy**](https://urldefense.com/v3/__http:/track.editorialmanager.com/CL0/http:*2F*2Fwww.plosone.org*2Fstatic*2Fpolicies.action*23sharing/1/010f0198589ea9bf-4a3b3287-a405-4b9f-9e33-748b4aace3f6-000000/8Nz12r9jCYM6cbOV4RUhN7miFtCQwyZDqTeAlbjMBKo=222__;JSUlJSU!!K-Hz7m0Vt54!mC6lNe6ONCWcoAg-r5MT7jR9b-xUfdMBEJIZCllyCv8-pkJD6SVeGcmdAAnUSG_dyey_Y2mVCrGYzEk$) **requires authors to make all data underlying the findings described in their manuscript fully available without restriction, with rare exception (please refer to the Data Availability Statement in the manuscript PDF file). The data should be provided as part of the manuscript or its supporting information, or deposited to a public repository. For example, in addition to summary statistics, the data points behind means, medians and variance measures should be available. If there are restrictions on publicly sharing data—e.g. participant privacy or use of data from a third party—those must be specified.**

**Reviewer #1: No**

**Reviewer #2: No**

All data and programs have been made available on GitHub. These provided data/programs include:

1. All R-code for the simulations
2. All data/programs for the diabetes case-study
3. All data/programs for analysis of the Boldt et al. trial which led to the retraction of 210 trials

**4. Is the manuscript presented in an intelligible fashion and written in standard English?

PLOS ONE does not copyedit accepted manuscripts, so the language in submitted articles must be clear, correct, and unambiguous. Any typographical or grammatical errors should be corrected at revision, so please note any specific errors here.**

**Reviewer #1: Yes**

**Reviewer #2: Yes**

5. Review Comments to the Author

**Please use the space provided to explain your answers to the questions above. You may also include additional comments for the author, including concerns about dual publication, research ethics, or publication ethics. (Please upload your review as an attachment if it exceeds 20,000 characters)**

**Reviewer #1: The authors propose an estimator for implausible study outcomes which is a commendable initiative. The approach is beyond comparing means and less subjective. Nevertheless, the manuscript is of a very general nature. The applied setting is intuitive, however, the findings might be supported by a simulation study with all underlying assumptions being varied (treatment effects, dropout rates, the set of trustworthy trials, etc.).**

We appreciate the helpful review and provided feedback, which we have incorporated into the new revision as detailed below.

Simulations were completed to the assess the impact of (i) heterogeneity of treatments effects, (ii) missing not at-random data, (iii) duplication of data rows, (iv) the randomization process (through the inclusion of null-effect simulations), and (v) the sampling of trials on the criteria for deeming a DiVBTA an outlier.

The simulation parameters for the HbA1c response were based on the Diabetes and Periodontal Therapy Trial (DPTT). This 30-million-dollar NIH-funded pivotal trial was published in JAMA and individual-level data was available (1). A gamma distribution was fitted to the 6-month HbA1c responses of the 514 participants. The R-code for this step together with its output are available on GitHub. We are not allowed to post the individual participant data of the DPTT trial on the GitHub page, but readers interested in duplicating this step can obtain the data from the DPTT investigator team.

Very important note: the simulations suggested that the variance formula for LnCVR published by Senior et al. were incorrect (2). Email communications with Dr. Senior confirmed that the variance formula published in 2020 (and which was adopted in the prior version of this manuscript) had errors. Corrections of these errors in the variance formula led to simulations with the expected type I error rate and led to the identification of 6 additional problematic trials in the diabetes case-study section.

We include a new Section 2 on simulation studies (pp. 9-13) and detailed below.

**Simulations to assess the impact of heterogeneous treatment effects on LnCVR**

Two R- programs on GitHub simulate how heterogeneity of treatment effects impact the natural logarithm of the Coefficient of Variation Ratio (LnCVR). Key aspects of the simulation are:

- Two clinical trial arms: A control group and a treatment group.
- Large (n=250 participants per trial arm) and small clinical trial (n=20 participants per trial arm) settings.
- Two parameters characterizing the heterogeneity of treatment effects:
  - The proportion of participants in the intervention group having a heterogeneous treatment effect (range 0% to 50% by 10% increments).
  - The size of the heterogeneous treatment effect. The size of the heterogeneous treatments was ranged between 0 and 1.4%. This range was based on a meta-analysis of 211 trials showing the 95% prediction intervals for HbA1c responses in clinical trials ranges between an HbA1c improvement of -1.00 to a worsening of Hba1c to 0.4.
- The simulation is repeated 1,000 times for (i) each size of heterogeneous treatment effect and (ii) proportion of participants to get reliable averages.

The impact of heterogeneous treatment effects on LnCVR was thus evaluated in 96 settings: combining the 6 specifications of the size of the subgroup (0%, 10%, 20%, 30%, 40%, 50%), the 8 distinct treatment effects in these subgroups (0.0%, 0.2%, 0.4%, 0.6%, 0.8%, 1%, 1.2%, 1.4%), and clinical trial size (large/small).

The LnCVR values, the % statistically significant LnCVR values, and the % statistically significant 3-sigma LnCVR values were calculated for each simulation and plotted as a function of (1) the treatment effect in the subgroup (0.0%,0.2%,0.4%,0.6%,0.8%,1%), and (2) the proportion of trial participants in the subgroup (10%, 20%, 30%, 40%, 50%) (Figure 2,3, and 4 for large samples and Figure 5,6, and 7 for small samples.

**Findings**

Large samples (250/trial arm) and HTE: The findings of the simulations show that large treatment effects (~ an improvement of 0.6% HbA1c) when present for 40% or more for the participants of the intervention lead to statistically significant LnCVRs that are small in magnitude (S1 Fig and S2 Fig). Important for this study, the heterogeneous treatment effects modeled in the simulations under no observable circumstances lead to statistically significant LnCVRs outliers (i.e., LnCVRs that that are large in magnitude or that exceed the 3-sigma bounds) (S3 Fig).

Small samples (n=20 per group) and HTE: Small sample sizes lead to two expected findings in the simulations: (1) the variability in the size of the LnCVR increases dramatically when compared to large sample sizes; unusually large LnCVR values become a common occurrence (S4 Fig), and (2) the probability to detect statistically significant LnCVR is small because the power of statistical tests to detect variance heterogeneity is small. The probability for HTE to cause statistically significant LnCVRs (S5 Fig) and statistically significant LnCVRs that cross the 3-sigma bounds (S6 Fig) is small.

**Simulations to assess the impact of duplication of trial participant responses.**

These simulations were initiated to assess whether statistically significant 3-sigma+ LnCVR would offer a tool to assess the duplication of rows of participants with excellent treatment responses. The R-programs simulating what happens if someone duplicates the best results in a clinical trial — specifically, the best responders (patients with the lowest HbA1c values) in the treatment group.

- Two clinical trial arms: A control group and a treatment group.
- Large (n=250 participants per trial arm) and small clinical trial (n=20 participants per trial arm) settings.
- The single best responder in the treatment group (lowest HbA1c) is copied and pasted multiple times into the dataset.
- Duplication levels tested: 0, 10, 20, 30, 40, or 50 copies of that one participant with the best response to treatment for the large sample trials, 0 to 4 copies for the small sample trials.
- To make room, the worst 10–50 patients (highest HbA1c) in the treatment group are removed.
- The simulation is repeated 1,000 times for each duplication level.

The aim was to mimic data where someone wants to make the treatment look more consistent (less variable) by inflating the number of excellent outcomes.

**Findings**

Large samples (250/trial arm) and data duplication: Twenty percent data duplications leads to 70% chance for a statistically significant LnCVR (S9 Fig). The probability of detecting statistically significant LnCVR outliers because of data duplication remains essentially zero.

Small samples (20/trial arm) and data duplication: Data duplications in small samples does not lead to statistically significant LnCVRs or statistically significant LnCVR outliers.

We additionally simulated not-missing-at-random data detailed in a response below.

**Major
- page 4, 1st paragraph: respective DIVBTA estimators should be mentioned in more detail. The paper could also benefit from benchmarking results of alternative estimates in the applied setting.**

Thank you for this suggestion to include respective DiVBTA estimators, we addressed this in three ways:

(1) criteria for the selection of DiVBTAs are now presented and three DiVBTA estimators fulfilling these criteria are presented in detail in the revised manuscript.

(2) an additional case-study, the Boldt trial, is presented illustrating an alternative DiVBTA measure: the logarithm of the variance ratio

(3) the consistency of 5 DiVBTA estimators for the HbA1c data (diabetes case-study) was evaluated below. The below graphic shows > 4-sigma outliers are consistently classified as outliers regardless of the DiVBTA estimator used. We elected not to present Coefficient of Variation Differences (CoV) or Differences of Variances (DoV) in the manuscript as ratio measures are preferable for outlier detect (p. 6).

**- page 4, 2nd paragraph: “The set of randomized controlled trials at the basis of clinical guidelines” and, in particular, the definition of a “set of trustworthy trials” should be extended. So far authors remain vague on this definition albeit it might have crucial relevance for application of DIVBTAs. Which criteria qualify to be part of the set, placebo-controlled trials, trials with active control (head-to-head comparisons), efficacy/safety trials, sample size of trials? When is the size of the set sufficient? How to flag a trial as untrustworthy in absence of such a set?**

Thank you for this comment and we agree more detail on the set of included trials was warranted.

We no longer use the term “trustworthy” trials. We had chosen this term because of the motto present next to the term “Cochrane Library”: “Trusted evidence. Informed decisions. Better health.” Because of this motto, we had referred to the trials present in Cochrane reviews as a set of trustworthy clinical trials.

Dr. Emma Sydenham, the second reviewer, provided the reasons why this label was not advisable and we have incorporated this information in the revised manuscript. The reasons to avoid “trustworthy” were:

1. Cochrane policies on how to handle concerns about the trustworthiness of publications were first published in 2021.
2. Various tools for checking trustworthiness of clinical trials prior to inclusion in Cochrane reviews appeared after 2021.
3. Implementing these changes to improve trustworthiness will take time.

We investigated the robustness of the method towards sampling trials in the diabetes case study. The 3-sigma prediction interval was robust to whether all trials were used to estimate the interval (226 trials with 289 LnCVR estimates) or just trials with non-problematic baseline data (175 trials with 229 LnCVR estimates). Bootstrapping of all trials (226 trials with 289 LnCVR estimates) showed that the 3-sigma bounds were remarkably robust towards which trials were included in their construction (S2 Table; included below).

We did 1,000 bootstrap samples of the 226 clinical trials we identified for the diabetes systematic review, that is, randomly resampling the 226 studies with replacement 1,000 times. Each time we ran the meta-analysis, calculated the average LnCVR, the 3-sigma bound, the median LnCVR per study, and the IQR-based outlier fences. The key results show the 3-sigma bounds were robust — the lower 3-sigma bound stays around −0.55, the upper 3-sigma bound stays around +0.48 -- the average LnCVR, median LnCVR, and the outlier fences remain similarly robust.

The following table presents the results of the bootstrapping:

| **Variable** | **Original** | **Bootstrap Mean** | **Bootstrap SE** | **95% CI** |
| --- | --- | --- | --- | --- |
| lower_lncvr3sigma | -0.579 | -0.565 | 0.055 | (-0.673, -0.468) |
| upper_lncvr3sigma | 0.531 | 0.519 | 0.067 | (0.395, 0.65) |
| LnCVR | -0.024 | -0.023 | 0.017 | (-0.053, 0.009) |
| median_LnCVR | -0.024 | -0.029 | 0.016 | (-0.063, 0) |
| fenceLnCVR_up.75% | 0.488 | 0.512 | 0.062 | (0.409, 0.651) |
| fenceLnCVR_low.25% | -0.567 | -0.589 | 0.056 | (-0.715, -0.503) |

The R-program used to conduct this bootstrap analysis is available on GitHub.

In the unlikely scenario in which there are no systematic reviews of clinical trials available, the proposed methods cannot be applied. We have added this as a limitation in the discussion (p. 17).

**- The first assumption “First, there is no treatment effect heterogeneity – the treatment effect is the same for every individual” I'm not sure if this is mentioned here as a more general or specific assumption for the estimator? This cannot apply to placebo-controlled studies. If otherwise, please comment.**

We thank the reviewer for this comment.

The selection of a DiVBTA estimator depends on whether the outcome variable has a normal distribution, whether the trial has two or more arms, and whether individual participant data are available. An assumption of no treatment effect heterogeneity is not considered for the selection of a DiVBTA estimator. The minimum requirements and assumptions for the selection were summarized by Mills et al. (3).

The methods can be applied to placebo-controlled studies. This comment is addressed in the following three points.

1. **Background:** The idea that heterogeneous treatment effects exist in placebo-controlled trials was first suggested by Fisher within the context of agricultural experiments (according to Senn) (4). The observed variability in the treatment arm consists of (i) a variation in response to treatment by patients (sometimes referred to as medication‐specific effects or treatment-specific effects) plus (ii) non-specific effects including regression to the mean, placebo effects, and random terms such as (but not restricted to) measurement error.  In the placebo arm, the medication‐specific effects or treatment-specific effects are absent which leads to the expectation that variability in response to treatment is expected to be larger than the variability in response to placebo.
2. **Published heterogeneity of treatment effects for placebo-controlled trials.** Several investigators have assessed heterogeneity of treatment effects for placebo-controlled trials. Table 1 below suggest that most studies have concluded that the hypothesis of homogeneous treatments effect for placebo-controlled trials cannot be rejected.

Table 1. Overview of studies assessing the presence of heterogeneous treatment in placebo-controlled trials

| Study | Sample | DIVBTA -endpoint | DIVBTA -change | Conclusion |
| --- | --- | --- | --- | --- |
| (5) | 222 RCTs; 19 different antidepressants compared with placebo; 345 comparisons, comprising a total of 61144 adults with an MDD diagnosis. | 0.98 (95% CI 0.96 to 1.00, I^2^ = 0%) | 1.00 (95% CI 0.99 to 1.02, I^2^ = 0%) | “cannot reject the null hypothesis of equal variances” |
| (6) | 163 randomised, placebo-controlled trials (51 396 patients) | VR=1.01 (CI 0.99 to 1.02) | CVR=0.82 (CI 0.80 to 0.84) | “The lack of increased treatment-outcome variance in the antidepressants versus placebo groups in randomised controlled trials indicates that no or only very small subgroups of patients respond particularly well to antidepressants” |
| (7) | 134 trials (total n = 46,646). | CVR = 0.94, (95% CI 0.93-0.95, p < .0001) |  | Our results instead suggest that antidepressants show a relatively uniform effect. |
| (8) | 169 randomized, controlled trials including 58,687 patients. |  |  | “The published data from RCTs on antidepressants for the treatment of major depression is compatible with a near-constant treatment effect. Although it is impossible to rule out a substantial treatment effect heterogeneity, its existence seems rather unlikely.” |
| (3) | 11 trials reporting  BDI outcomes, varying in size from 44 to 216 participants. |  |  | There was no  strong evidence of heterogeneity in the difference in variances  meta-analysis (I2 = 20%). |
| (3) | The 22 trials in the meta-analysis reporting the effect of statins versus placebo or no treatment on LDL cholesterol,34  varied in size from 199 to 374 total participants |  |  | There was no strong evidence of heterogeneity  in the DiV meta-analysis (I2 = 36.5%). |
| (9) | 7 trials varied in sample size from 63 to 307 |  |  | Heterogeneity of treatment effects “Our  results suggest that whilst LC diets are effective in a large proportion of the population, for a subset of  individuals, calorie restricted diets may be more effective. “ |
| (10) | 18 studies that included 965 patients | VR of 0.99 (95% CI:0.87–1.14) | 0.97 (95% CI 0.82–1.14) | “Our analysis did not support the existence of a heterogeneous patient-by-patient response to NSBBs in cirrhosis.” |

3. **Heterogeneity of treatment effects for placebo-controlled diabetes trials.** The Cochrane description for the comparator and the intervention for each of the 305 included trials was abstracted (data available on https://github.com/mhujoel/DIVBTA/). The heterogeneity of treatment effects was assessed for the 18 placebo-controlled diabetes trials (19 LnCVR estimates). The results show that the hypothesis of homogeneity of variance failed to be rejected (LnCVR= -0.0425; Se(LnCVR)= 0.0747; t-value=-0.569; p-value= 0.577).

**Please specify in more detail the second assumption “Second, the error of the outcome does not depend on the treatment or the outcome”. How is error defined?** **Is that a plausible assumption for all kinds of outcomes?** **For example, single- or double-bounded outcomes should be considered, please see:** [**https://doi.org/10.3102/1076998610396895**](https://urldefense.com/v3/__https:/doi.org/10.3102/1076998610396895__;!!K-Hz7m0Vt54!mC6lNe6ONCWcoAg-r5MT7jR9b-xUfdMBEJIZCllyCv8-pkJD6SVeGcmdAAnUSG_dyey_Y2mVCiZ4aJM$). **Variances should also change, in particular, when a** **considerable proportion of patients achieve remission.**

A statistical model (e.g., ANOVA) is fitted to outcome data (e.g., HbA1c). This fitted model allows computing residuals (errors) for each participant. These residuals should be independent (uncorrelated) to both treatment assignment (diabetic intervention) and the outcome (e.g., observed HbA1c outcome).

As reported by Mills et al., “*differences in variance can be caused by many factors*”. Single- or double-bounded outcomes is indeed one reason variances can be unequal across treatment arms. This possibility was mentioned in the report as follows: “ceiling or floor effects if the outcome measure has practical limits (e.g., a scale with a maximum or minimum value)” and remains mentioned in the current report (p. 18 and included below).

Empirical evidence suggests the impact of floor or ceiling effects is limited. Three meta-analyses of variances in Table 1 above are based on the Hamilton Depression Rating Scale (HAM-D), which has a theoretical floor of 0 and ceiling of 52–75. These three meta-analyses fail to reject the hypothesis of homogeneity of variances (5, 6, 8).

We have included that variances may differ when a considerable proportion of patients achieve remission in the discussion (p. 18 and included below).

*When investigating significant differences in variance between trial arms, many potential explanations must be considered. These include floor or ceiling effects, the occurrence of complete remission, and other factors.*

**- Please also be more detailed about randomization. The phrasing “A particularly unlucky randomization may lead to …” appears not well aligned with common terminology.**

The impact of randomization on DiVBTAs was extensively addressed with simulations.

All simulations included the null-effect (e.g., heterogenous treatment effect size set to 0, missing not-at-random prevalence set to 0, and number of duplicated data lines included set to 0). These simulations show the impact of randomization on DiVBTAs. Randomization can by accident lead to imbalances in confounders leading to significant DiVBTAs. All simulations show that, as expected, 5% of the DiVBTA will be statistically significant (p < 0.05) by chance (S2 Fig, S5 Fig, S7 Fig, S8 Fig, S9 Fig, S10 Fig). In simulations with large sample sizes, while randomization led to 5% of DiVBTAs being statistically significant, none were statistically significant DiVBTA **outliers**. In simulations with small sample sizes, randomization led to ~5% of DiVBTAs being statistically significant outliers.

**- The phrasing “the variability of DIVTBA can be impacted by the noise created by participant dropout” is unclear. In which form do dropouts add noise? To this reviewers experience, they introduce imbalanced patient characteristics and missingness. In case of selective dropout this leads to upwards/downwards biased results.**

Two R- programs (available on https://github.com/mhujoel/DIVBTA/) simulate how dropouts in a clinical trial affect the natural logarithm of the Coefficient of Variation Ratio (LnCVR).

Simulation details:

- Two clinical trial arms: A control group and a treatment group.
- Large (n=250 participants per trial arm) and small clinical trial (n=20 participants per trial arm) settings.
- Dropouts occur in the control group, and are not random — the worst responders (people with the highest values of a biomarker called "A1c") are the ones who drop out. This extreme type of "Missing Not At Random" (MNAR) was selected to provide a robust assessment.
- Dropout rates tested: 0%, 10%, 20%, 30%, 40%, and 50% of the control group.
- The simulation is repeated 1,000 times for each dropout level.

The impact of heterogeneous treatment effects on LnCVR was thus evaluated in 6 settings (0%, 10%, 20%, 30%, 40%, 50%). The LnCVR values, the % statistically significant LnCVR values, and the % statistically significant 3-sigma LnCVR values were calculated for each simulation and plotted as a function of dropouts for small and large trials (Figure S7 and S8).

We have added a discussion of these simulations in the Section 2 (pp. 9-13) and provide a summary of the findings below:

Large samples (250/trial arm) and MNAR: Simulations show that the magnitude and statistical significance of LnCVR are extremely sensitive to informative dropout. An informative dropout rate as small as 10% led 80% of the LnCVR values to be statistically significant. An informative dropout rate of 20% led to 100% of the LnCVR to be statistically significant. Of relevance to this report, the probability of detecting statistically significant 3-sigma LnCVR outliers was trivial for a dropout rate of up to and including 30% (S7 Fig). The probability of detecting statistically significant 3-sigma LnCVR outliers rose to ~ 25% and ~60%, when the highly informative dropout rate increased to 40% and 50% respectively. In conclusion, statistically significant LnCVR outliers can be a marker for highly informative dropout rates of 40% or larger.

Small samples (20/trial arm) and MNAR: The variability of simulated LnCVR values increases substantially in small sample situations as expected. As the MNAR increases, the probability to detect statistically significant LnCVRs and the probability to detect statistically significant LnCVR outliers stays below 40%.

**- The whole paragraph at the end of page 5/1st page 6 is unclear.**

This paragraph was deleted, we apologize for the lack of clarity.
 **- The section “Interpretation of the DIVBTA variance observed in a set of trustworthy trials” might benefit from a table summarizing the assumptions for DIVBTA such as expectations regarding the mean/variability in trustworthy vs. untrustworthy trials, impact of dropout, interaction, by chance, trial size, randomization, and further biases.**

The term “trustworthy trials” is no longer used. Simulations now show the impact of dropout, heterogeneous treatment effects, trial size, the randomization process (through the inclusion of null-effect simulations), and trial selection (through bootstrapping of the DiVBTA outlier criteria for the diabetes case study).

**Minor**
**Abstract: Please correct typos “. to illustrate this approach, we assessed the DIVBTAs for Hemoglobin A1cin a systematic sample”**

The sentence was corrected. “To illustrate this approach, we assessed the DIVBTAs for Hemoglobin A1c in a systematic sample of 224 diabetes trials with 42,780 participants included in a systematic sample of 35 Cochrane reviews published between 2010 and 2023.”

**Please revise the use and setting of references with/without leading spaces.**

The manuscript was revised to a consistent use of references without leading spaces

**Reviewer #2: I very much enjoyed reading your manuscript and look forward to seeing it published soon.
With kind regards, Emma Sydenham (Senior Editor, Cochrane Database of Systematic Reviews)**

We thank the reviewer for their kind comments and appreciate all the provided feedback, which we have incorporated into the revision as detailed below.

**Major comments:**

**1) Concerning point 3 above 'Have the authors made all data underlying the findings of their manuscript fully available' I have answered no as the list of reviews identified in 'II. Problematic differences in variance between trial arms (DIVBTA): Application' is not given. It would be helpful if the thirty-five Cochrane reviews that were identified and reported HbA1c could be referenced in this section, or listed in a table.**

All data presented in the manuscript are now available on GitHub (https://github.com/mhujoel/DIVBTA/). The file containing all data also includes the bibliographic references of the 35 Cochrane review and 305 unique clinical trials included in the 35 Cochrane review. In addition to the data, the GitHub website also includes the statistical programs to reproduce the diabetes case-report, the Boldt case report (new), and the simulations presented in the manuscript.

**Minor comments:**
**1) The third sentence of the introduction does not follow. I suggest re-phrasing based on the following comment, and those below. While it is true that the unreliable trials have permeated clinical guidelines, I disagree that this has contributed to a growing crisis in research integrity.**

**Rather, unreliable trials have permeated guidelines because research integrity and regulatory compliance criteria were not previously included in meta-analysis and guideline development processes. This is partly due to the information not being requested or made available by publishers in previous decades, and the issue not having been taken up by a working group in evidence based medicine years ago (i.e. in the early 2000s). I don't think there is a growing crisis in research integrity - I think research integrity was never raised as an issue until recently.**

**Research integrity issues were highlighted during the COVID pandemic in particular, in 2020, which was some 30 years after evidence based medicine started taking hold as an academic discipline. I think the possibility of tackling research integrity issues in evidence based medicine has never been better than at present, because now there are a handful of research integrity checklists, policies, and methods (such as this paper) which are available for researchers to use which didn't previously exist.**

**Also, looking back ten years, the critics of research integrity were not morally wrong or factually incorrect in their views. (Here I refer to an old debate (**[**https://www.bmj.com/content/350/bmj.h2463**](https://urldefense.com/v3/__https:/www.bmj.com/content/350/bmj.h2463__;!!K-Hz7m0Vt54!mC6lNe6ONCWcoAg-r5MT7jR9b-xUfdMBEJIZCllyCv8-pkJD6SVeGcmdAAnUSG_dyey_Y2mV7b7Fcmw$)**) and a more recent commentary (**[**https://www.jclinepi.com/article/S0895-4356(25)00003-4/pdf**](https://urldefense.com/v3/__https:/www.jclinepi.com/article/S0895-4356(25)00003-4/pdf__;!!K-Hz7m0Vt54!mC6lNe6ONCWcoAg-r5MT7jR9b-xUfdMBEJIZCllyCv8-pkJD6SVeGcmdAAnUSG_dyey_Y2mVe9wQFtk$)**).) Clinical trials are highly regulated and so it should indeed be safe to assume that they were conducted according to the relevant domestic and international regulations. It should be the case that study reports accurately reflect lawfully collected clinical trial data. One of a number of problems is that new computing technologies have been developed which make it easy to generate fake data, and these new technologies have become more accessible over the last ten years. Multiple issues relating to computer generated data, changes in medical journal publishing, trial registration, international harmonisation of clinical trial conduct and electronic data collection exist in parallel and are constantly evolving. The more recent acknowledgement that research integrity can be a problem, and the use of solutions, are making evidence based clinical guidelines safer. Here is one such example:** [**https://www.cochrane.org/about-us/news/cochrane-launches-new-feature-identify-retracted-publications**](https://urldefense.com/v3/__https:/www.cochrane.org/about-us/news/cochrane-launches-new-feature-identify-retracted-publications__;!!K-Hz7m0Vt54!mC6lNe6ONCWcoAg-r5MT7jR9b-xUfdMBEJIZCllyCv8-pkJD6SVeGcmdAAnUSG_dyey_Y2mVlAFD9Fw$) **So I would encourage you to reconsider using the phrase 'a growing crisis in research integrity'.**

Thank you for these points – the reference to 'a growing crisis in research integrity' was deleted and replaced with suggested content in the Introduction (p. 3) and included below.

*“Problematic clinical trials are widespread and erode the credibility of health information. It is estimated that 25% of published clinical trials may be flawed or fraudulent (11). In absolute terms, hundreds of thousands of trials are believed to lack credibility (12). These unreliable trials have permeated meta-analyses and clinical guidelines (13-16),* ***which has led to concerted efforts at curbing their influence.***

*Global regulatory guidelines by the International Council for Harmonisation Good Clinical Practice have imposed a series of legally binding or quasi-binding requirements on data collection that are designed to minimize fraud, misrepresentation, and data integrity issues (17). Implementation of these guidelines by organizations such as the Food and Drug Administration can lead to disqualification proceedings against clinical investigators (18). Independently, starting in 2020, coinciding with fraudulent COVID-19 data (19), increasing efforts became aimed at eliminating potentially problematic trials from systematic reviews. A 2021 Cochrane editorial warned of the threat posed by untrustworthy or "problematic" studies, highlighting that retracted studies are only the tip of the iceberg. The Cochrane editorial coincided with the release of new Cochrane guidelines on how to handle concerns about the trustworthiness of a publication when no formal post-publication correction exists (20). Checklists to identify problematic trials (21-24) and recommendations on how these tools should become integrated during research synthesis followed (25).*

**2) The following sentence 'A 2021 Cochrane editorial...' could be expanded. This editorial by Boughton, Wilkinson and Bero was the announcement of Cochrane's Editorial Policy on Managing Potentially Problematic Studies, which was developed over 5 years with broad consultation. I assume I have been invited to comment on your work as I am listed as a member of the Policy advisory committee. Unfortunately the Policy document does not have its own DOI, because it is included in an online policy manual and website, so the editorial is often referenced instead of the policy implementation guidance web link. The editorial you reference was only one means of disseminating the policy, there are also training materials produced by Cochrane, there was a popular blog post by Richard Smith 'Time to assume that health research is fraudulent until proven otherwise?'** [**https://blogs.bmj.com/bmj/2021/07/05/time-to-assume-that-health-research-is-fraudulent-until-proved-otherwise/**](https://urldefense.com/v3/__https:/blogs.bmj.com/bmj/2021/07/05/time-to-assume-that-health-research-is-fraudulent-until-proved-otherwise/__;!!K-Hz7m0Vt54!mC6lNe6ONCWcoAg-r5MT7jR9b-xUfdMBEJIZCllyCv8-pkJD6SVeGcmdAAnUSG_dyey_Y2mV1g1aizU$) **, among other resources and conference round table discussions.
However, rather than referring to prior calls for action, you could present the work as a contribution to the other new developments which have followed. For example, a research integrity tool with an associated R package was developed by Hunter et al:** [**https://doi.org/10.1002%2Fjrsm.1738**](https://urldefense.com/v3/__https:/doi.org/10.1002*2Fjrsm.1738__;JQ!!K-Hz7m0Vt54!mC6lNe6ONCWcoAg-r5MT7jR9b-xUfdMBEJIZCllyCv8-pkJD6SVeGcmdAAnUSG_dyey_Y2mVfDsdcks$) **and there is also an R package for the statistical checks of the REAPPRAISED checklist:** [**https://reappraised.wordpress.com/2023/03/28/the-reappraised-r-package/**](https://urldefense.com/v3/__https:/reappraised.wordpress.com/2023/03/28/the-reappraised-r-package/__;!!K-Hz7m0Vt54!mC6lNe6ONCWcoAg-r5MT7jR9b-xUfdMBEJIZCllyCv8-pkJD6SVeGcmdAAnUSG_dyey_Y2mVMAzwUHM$) **Ideally this piece of work will inform new R packages, which are currently being used to automate research integrity checks in the field of Data Science.**

**Are you aware that an R package has already been developed for this analysis? It might be worth referencing, too:** [**https://github.com/harrietlmills/DetectingDifferencesInVariance**](https://urldefense.com/v3/__https:/github.com/harrietlmills/DetectingDifferencesInVariance__;!!K-Hz7m0Vt54!mC6lNe6ONCWcoAg-r5MT7jR9b-xUfdMBEJIZCllyCv8-pkJD6SVeGcmdAAnUSG_dyey_Y2mVLdQp9as$)

Thank you for pointing out these needed changes. The suggested references have been added to the Introduction (pp. 3-4) and included below.

*Trials flagged as having highly unusual distributions when compared to those expected by chance have shown a higher likelihood of retraction (15-17). Additional statistical methods have become increasingly available and at least two statistical packages integrate methods to re-appraise the publication integrity in groups of randomized controlled trials (18-21).*

The study of Mills et al. which refers to the referenced R-package was previously cited and remains cited (previously reference #14; now reference #21).

**3) You have chosen to examine a cohort of Cochrane reviews for your example. The analysis that you have done for these trials is good, I just think there is a slight problem in the way you have explained the rationale for selecting these trials which should be reconsidered.** **The search for reviews starts in 2010 which is prior to the development of Cochrane's Editorial Policy for Managing Potentially Problematic Studies. So the trials that are included in your analysis are not ones which went through a Trustworthiness Screening Tool (such as: Identifying and handling potentially untrustworthy trials – Trustworthiness Screening Tool (TST). Developed by the Cochrane Pregnancy and Childbirth Group. Alfirevic Z, Kellie FJ, Weeks J, Stewart F, Jones L, Hampson L, on behalf of the Pregnancy and Childbirth Editorial Board and 10.1002/cesm.12037). The content of your work is fine and should be published, I just think the way you have framed the issue of the reviews being trustworthy because they are Cochrane reviews is not quite right if those reviews were published prior to Cochrane's policy and the reviews didn't incorporate a trustworthiness screening tool or another research integrity assessment tool or strategy.**

Thank you; the suggested corrections were incorporated in Section 4 (p. 16) and included below.

*The high prevalence of potentially problematic trials reported here (~9%) reflects on calendar years when awareness and scrutiny of problematic trials was low. Included Cochrane reviews were published starting in 2010; all but one of the reviews included were published before Cochrane’s 2021 editorial policy on managing potentially problematic studies. Authors of even the most recent (2022) Cochrane reviews may have been unaware of the 2021 editorial policy, in part because it exists separately from the Cochrane Handbook.*

**4) Further to point 3) with regards to the paragraph 'Trustworthy trials as a source of DIVBTA estimates' (p.4), 'By focusing on a set of trials viewed as trustworthy by an authoritative organization' (p.6), and 'A non-parametric approach to define unusual DIVBTAs...' (p.7) it's worth noting that not all Cochrane authors are aware of the** **Policy on Managing Potentially Problematic Trials. This is partly due to the fact that the Cochrane Handbook is long, and the Policy is described in a separate online manual covering Cochrane's editorial policies. Even at present (July 2025), not every trial included in a Cochrane review is assessed using a research integrity tool. I have no doubt that will come in the future, but we aren't there yet and very few trials were statistically checked in the past. Furthermore, teaching about meta-analysis varies in scope and research integrity may not be included in the curriculum. Research integrity was not commonly taught prior to 2020, until the COVID pandemic brought issues concerning research integrity into public discourse.**

Thank you, these points were included in Section 4 (p. 16) and again included below.

*The high prevalence of potentially problematic trials reported here (~9%) reflects on calendar years when awareness and scrutiny of problematic trials was low. Included Cochrane reviews were published starting in 2010; all but one of the included reviews were published before Cochrane’s 2021 editorial policy on managing potentially problematic studies. Authors of even the most recent (2022) Cochrane reviews may have been unaware of the 2021 editorial policy, in part because it exists separately from the Cochrane Handbook.*

**5) I encourage you to re-phrase the sentences 'A non-parametric approach to define unusual DIVBTAs is to derive the median DIVBTA for each trustworthy trial' (p.7) and 'The two criteria for defining the set of trustworthy clinical trials were...' (p.10). I understand what you mean; however, I would like to point out that the trials you have selected for analysis have not been through a formal trustworthiness assessment prior to publication of the Cochrane reviews. The only Cochrane editorial group that routinely assessed all studies for trustworthiness prior to inclusion in the review was the Pregnancy and Childbirth Group, which developed and applied their Cochrane PCG-TST tool in the reviews for which they held editorial responsibility. (Note, this has all changed now as there is a 'new' Cochrane Central Editorial Service.) So while your work presented in this paper is true and valid in terms of the actual analysis, it is not the case that the studies included in the 35 Cochrane reviews were assessed as being trustworthy to start with. No trustworthiness assessment was done, apart from possibly filtering out the retracted studies as part of the searching procedures (as per the Mandatory standard C48 Examining Errata, Cochrane Handbook section 4.4.6 and the technical supplement section 3.9). The work you have done and presented in this paper is good and should be published, I'm just not sure you should say that the trials are trustworthy if they have not been through a trustworthiness screening tool or a research integrity checklist (such as PCG-TST, the Reappraised checklist (Grey et al), RIA (Weibel et al), TRACT (Mol et al), or a procedure as described in the RIGID Framework (Mousa et al)). These research integrity tools hadn't been developed in 2010 which is the start date of your search.**

Thank you. The reference to “trustworthy trials” has been deleted. We had chosen this term because of the motto present next to the term “Cochrane Library”: “Trusted evidence. Informed decisions. Better health.”

The manuscript now reports how Cochrane policies for handling concerns about the trustworthiness of publications were first published in 2021, how various tools for checking trustworthiness of clinical trials prior to inclusion in Cochrane reviews appeared after 2021, and how fully implementing these changes to improve trustworthiness will take time (Section 4 p. 16).

Instead of referring to “trustworthy trials”, we have now investigated to what extent the methods to identify potentially problematic trials depends on the selection of randomized controlled trials.

We investigated the robustness of the method towards sampling trials in the diabetes case study. The 3-sigma prediction interval was robust to whether all trials were used to estimate the interval (226 trials with 289 LnCVR estimates) or just trials with non-problematic baseline data (175 trials with 229 LnCVR estimates). Bootstrapping of all trials (226 trials with 289 LnCVR estimates) showed that the 3-sigma bounds were remarkably robust towards which trials were included in their construction (S2 Table; included below).

We did 1,000 bootstrap samples of the 226 clinical trials we identified for the diabetes systematic review, that is, randomly resampling the 226 studies with replacement 1,000 times. Each time we ran the meta-analysis, calculated the average LnCVR, the 3-sigma bound, the median LnCVR per study, and the IQR-based outlier fences. The key results show the 3-sigma bounds were robust — the lower 3-sigma bound stays around −0.55, the upper 3-sigma bound stays around +0.48 -- the average LnCVR, median LnCVR, and the outlier fences remain similarly robust.

The following table presents the results of the bootstrapping:

| **Variable** | **Original** | **Bootstrap Mean** | **Bootstrap SE** | **95% CI** |
| --- | --- | --- | --- | --- |
| lower_lncvr3sigma | -0.579 | -0.565 | 0.055 | (-0.673, -0.468) |
| upper_lncvr3sigma | 0.531 | 0.519 | 0.067 | (0.395, 0.65) |
| LnCVR | -0.024 | -0.023 | 0.017 | (-0.053, 0.009) |
| median_LnCVR | -0.024 | -0.029 | 0.016 | (-0.063, 0) |
| fenceLnCVR_up.75% | 0.488 | 0.512 | 0.062 | (0.409, 0.651) |
| fenceLnCVR_low.25% | -0.567 | -0.589 | 0.056 | (-0.715, -0.503) |

The R-program used to conduct this bootstrap analysis is available on GitHub.

**6) In the first paragraph of the Discussion, you could make reference to some other work in this area. For example, in order to understand the errors identified, the RIGID Framework and the Cochrane Policy recommend contacting trial authors to request clarification of the reasons for possible errors.
RIGID Framework:** [**https://www.thelancet.com/pdfs/journals/eclinm/PIIS2589-5370(24)00296-7.pdf**](https://urldefense.com/v3/__https:/www.thelancet.com/pdfs/journals/eclinm/PIIS2589-5370(24)00296-7.pdf__;!!K-Hz7m0Vt54!mC6lNe6ONCWcoAg-r5MT7jR9b-xUfdMBEJIZCllyCv8-pkJD6SVeGcmdAAnUSG_dyey_Y2mVwMtRnK0$) **Cochrane Policy:** [**https://www.cochranelibrary.com/cdsr/editorial-policies/problematic-studies-implementation-guidance**](https://urldefense.com/v3/__https:/www.cochranelibrary.com/cdsr/editorial-policies/problematic-studies-implementation-guidance__;!!K-Hz7m0Vt54!mC6lNe6ONCWcoAg-r5MT7jR9b-xUfdMBEJIZCllyCv8-pkJD6SVeGcmdAAnUSG_dyey_Y2mVzE8b_88$) **Very sadly there have been a few cases internationally of researchers taking their own life after their work was found to have problems, and one of the reasons for liaising with them is to make them aware their work is under review and to give them an opportunity to explain any problems that might be identified. (You can look up the case of Yoshiki Sasai, for example, which was highly publicised. However, there are other cases which have received no publicity so the actual number of cases is slightly higher than one can find in a web search.)**

Thank you. The suggestion is now incorporated in the Discussion (p. 16) and included below.

*The DiVBTA method, following current guidelines, could lead to the step of contacting the authors to clarify unusual standard deviations (25, 26). Alternatively, the DIVBTA methods could become integrated into checklists to assess the trustworthiness of clinical trial reports.*

**7) Your example is in diabetes research, but you could also reference where a similar analysis has been used in other areas of medicine, such as:** [**https://doi.org/10.1097/EDE.0000000000001401**](https://urldefense.com/v3/__https:/doi.org/10.1097/EDE.0000000000001401__;!!K-Hz7m0Vt54!mC6lNe6ONCWcoAg-r5MT7jR9b-xUfdMBEJIZCllyCv8-pkJD6SVeGcmdAAnUSG_dyey_Y2mVwJWdKGQ$) **and** [**https://doi.org/10.1002/bimj.202200116**](https://urldefense.com/v3/__https:/doi.org/10.1002/bimj.202200116__;!!K-Hz7m0Vt54!mC6lNe6ONCWcoAg-r5MT7jR9b-xUfdMBEJIZCllyCv8-pkJD6SVeGcmdAAnUSG_dyey_Y2mVpmC3Ia8$) **among others.**

Thank you. Both references were included in the manuscript (previously reference #14 and #16) and both references remain included in the revised manuscript.

6. PLOS authors have the option to publish the peer review history of their article ([what does this mean?](https://urldefense.com/v3/__https:/track.editorialmanager.com/CL0/https:*2F*2Fjournals.plos.org*2Fplosone*2Fs*2Feditorial-and-peer-review-process*23loc-peer-review-history/1/010f0198589ea9bf-4a3b3287-a405-4b9f-9e33-748b4aace3f6-000000/ztGvb3DZJ1yiamWRJljSny0ElDaZUUJNY0N98F_gLrk=222__;JSUlJSUl!!K-Hz7m0Vt54!mC6lNe6ONCWcoAg-r5MT7jR9b-xUfdMBEJIZCllyCv8-pkJD6SVeGcmdAAnUSG_dyey_Y2mVKwKueVA$)). If published, this will include your full peer review and any attached files.


If you choose “no”, your identity will remain anonymous but your review may still be made public.


**Do you want your identity to be public for this peer review?** For information about this choice, including consent withdrawal, please see our [Privacy Policy](https://urldefense.com/v3/__https:/track.editorialmanager.com/CL0/https:*2F*2Fwww.plos.org*2Fprivacy-policy/1/010f0198589ea9bf-4a3b3287-a405-4b9f-9e33-748b4aace3f6-000000/pwWLFloqS0eIzpxO2fjBeyJghubAW869vNh4sfazxmc=222__;JSUl!!K-Hz7m0Vt54!mC6lNe6ONCWcoAg-r5MT7jR9b-xUfdMBEJIZCllyCv8-pkJD6SVeGcmdAAnUSG_dyey_Y2mVEE5nmUg$).

Reviewer #1: **Yes:** Dr. Adrian Richter

Reviewer #2: **Yes:** Emma Sydenham (Senior Editor, Cochrane Database of Systematic Reviews)

[NOTE: If reviewer comments were submitted as an attachment file, they will be attached to this email and accessible via the submission site. Please log into your account, locate the manuscript record, and check for the action link "View Attachments". If this link does not appear, there are no attachment files.]

While revising your submission, please upload your figure files to the Preflight Analysis and Conversion Engine (PACE) digital diagnostic tool, [https://pacev2.apexcovantage.com/](https://urldefense.com/v3/__https:/track.editorialmanager.com/CL0/https:*2F*2Fpacev2.apexcovantage.com*2F/1/010f0198589ea9bf-4a3b3287-a405-4b9f-9e33-748b4aace3f6-000000/gJCEjx6BvWYTZGcrrBtS2FbsgbQ-Um7J9W4se4APiJE=222__;JSUl!!K-Hz7m0Vt54!mC6lNe6ONCWcoAg-r5MT7jR9b-xUfdMBEJIZCllyCv8-pkJD6SVeGcmdAAnUSG_dyey_Y2mVF9iZSIE$). PACE helps ensure that figures meet PLOS requirements. To use PACE, you must first register as a user. Registration is free. Then, login and navigate to the UPLOAD tab, where you will find detailed instructions on how to use the tool. If you encounter any issues or have any questions when using PACE, please email PLOS at [figures@plos.org](mailto:figures@plos.org). Please note that Supporting Information files do not need this step.

1.	Engebretson SP, Hyman LG, Michalowicz BS, Schoenfeld ER, Gelato MC, Hou W, et al. The effect of nonsurgical periodontal therapy on hemoglobin A1c levels in persons with type 2 diabetes and chronic periodontitis: a randomized clinical trial. Jama. 2013;310(23):2523-32.

2.	Senior AM, Viechtbauer W, Nakagawa S. Revisiting and expanding the meta-analysis of variation: The log coefficient of variation ratio. Res Synth Methods. 2020;11(4):553-67.

3.	Mills HL, Higgins JPT, Morris RW, Kessler D, Heron J, Wiles N, et al. Detecting Heterogeneity of Intervention Effects Using Analysis and Meta-analysis of Differences in Variance Between Trial Arms. Epidemiology. 2021;32(6):846-54.

4.	Senn S. Mastering variation: variance components and personalised medicine. Stat Med. 2016;35(7):966-77.

5.	Munkholm K, Winkelbeiner S, Homan P. Individual response to antidepressants for depression in adults-a meta-analysis and simulation study. PloS one. 2020;15(8):e0237950.

6.	Ploderl M, Hengartner MP. What are the chances for personalised treatment with antidepressants? Detection of patient-by-treatment interaction with a variance ratio meta-analysis. BMJ Open. 2019;9(12):e034816.

7.	Guo X, McCutcheon RA, Pillinger T, Mizuno Y, Natesan S, Brown K, et al. The magnitude and heterogeneity of antidepressant response in depression: A meta-analysis of over 45,000 patients. J Affect Disord. 2020;276:991-1000.

8.	Volkmann C, Volkmann A, Muller CA. On the treatment effect heterogeneity of antidepressants in major depression: A Bayesian meta-analysis and simulation study. PloS one. 2020;15(11):e0241497.

9.	Senior AM, Gosby AK, Lu J, Simpson SJ, Raubenheimer D. Meta-analysis of variance: an illustration comparing the effects of two dietary interventions on variability in weight. Evol Med Public Health. 2016;2016(1):244-55.

10.	Alsaeid M, Sung S, Bai W, Tam M, Wong YJ, Cortes J, et al. Heterogeneity of treatment response to beta-blockers in the treatment of portal hypertension: A systematic review. Hepatol Commun. 2024;8(2).

11.	Van Noorden R. Medicine is plagued by untrustworthy clinical trials. How many studies are faked or flawed? Nature. 2023;619(7970):454-8.

12.	Ioannidis JPA. Hundreds of thousands of zombie randomised trials circulate among us. Anaesthesia. 2021;76(4):444-7.

13.	Anonymous. There is a worrying amount of fraud in medical research And a worrying unwillingness to do anything about it,. The Economist. 2023.

14.	Blake H, Watt H, Winnett R. Millions at risk in drug fraud scandal: INVESTIGATION prosecutors investigate doctor. The Daily Telegraph. 2011 March 4th, 2011.

15.	Subbaraman N. The band of debunkers busting bas scientists; Stanford's president and a high-profile physicist are among those taken down by a growing wave of volunteers who expose faulty or fraudulent research. Wall Street Journal. 2023 Sep 24,2023.

16.	McKie R. Peer review and scientific publishing "The situation has become appalling': fake scientific papers push research credibility to crisis point. The Guardian. 2024 Feb 3, 2024

17.	International Council for Harmonisation of Technical Requirements for Pharmaceuticals for Human Use (ICH). Guideline for Good Clinical Practice E6(R2) 2016 [Available from: <https://www.ema.europa.eu/en/ich-e6-good-clinical-practice-scientific-guideline>.

18.	Food and Drug Administration. Clinical Investigators - Disqualification Proceedings 2016 [Available from: <https://www.fda.gov/inspections-compliance-enforcement-and-criminal-investigations/compliance-actions-and-activities/clinical-investigators-disqualification-proceedings>.

19.	Baker N, Van Noorden R, Maxmen A. Coronapod: The Surgisphere scandal that rocked coronavirus drug research. Nature. 2020.

20.	Boughton SL, Wilkinson J, Bero L. When beauty is but skin deep: dealing with problematic studies in systematic reviews. The Cochrane database of systematic reviews. 2021;6(6):ED000152.

21.	Hunter KE, Webster AC, Clarke M, Page MJ, Libesman S, Godolphin PJ, et al. Development of a checklist of standard items for processing individual participant data from randomised trials for meta-analyses: Protocol for a modified e-Delphi study. PloS one. 2022;17(10):e0275893.

22.	Mol BW, Lai S, Rahim A, Bordewijk EM, Wang R, van Eekelen R, et al. Checklist to assess Trustworthiness in RAndomised Controlled Trials (TRACT checklist): concept proposal and pilot. Res Integr Peer Rev. 2023;8(1):6.

23.	Weibel S, Popp M, Reis S, Skoetz N, Garner P, Sydenham E. Identifying and managing problematic trials: A research integrity assessment tool for randomized controlled trials in evidence synthesis. Res Synth Methods. 2023;14(3):357-69.

24.	Wilkinson J, Heal C, Antoniou GA, Flemyng E, Ahnstrom L, Alteri A, et al. Assessing the feasibility and impact of clinical trial trustworthiness checks via an application to Cochrane Reviews: Stage 2 of the INSPECT-SR project. Journal of clinical epidemiology. 2025;184:111824.

25.	Mousa A, Flanagan M, Tay CT, Norman RJ, Costello M, Li W, et al. Research Integrity in Guidelines and evIDence synthesis (RIGID): a framework for assessing research integrity in guideline development and evidence synthesis. EClinicalMedicine. 2024;74:102717.

26.	Cochrane. Policy for managing potentially problematic studies: implementation guidance. Chichester, UK: John Wiley & Sons. ; 2024 [Available from: <https://www.cochranelibrary.com/cdsr/editorial-policies#problematic-studies>.
