# Supplementary material for: Unusual outcome variances as a method to identify potentially problematic clinical trials
Source: PLoS One. 2026 Apr 15;21(4):e0346238. doi: 10.1371/journal.pone.0346238 (PMC13082665; doi:10.1371/journal.pone.0346238)
Supplement: Attachment — Submitted filename: response to reviewers_PH.docx [file pone.0346238.s011.docx]

Reviewers' comments:

Reviewer's Responses to Questions

**Comments to the Author**

1. If the authors have adequately addressed your comments raised in a previous round of review and you feel that this manuscript is now acceptable for publication, you may indicate that here to bypass the “Comments to the Author” section, enter your conflict of interest statement in the “Confidential to Editor” section, and submit your "Accept" recommendation.

Reviewer #1: (No Response)

Reviewer #2: All comments have been addressed

2. Is the manuscript technically sound, and do the data support the conclusions?

The manuscript must describe a technically sound piece of scientific research with data that supports the conclusions. Experiments must have been conducted rigorously, with appropriate controls, replication, and sample sizes. The conclusions must be drawn appropriately based on the data presented.

Reviewer #1: Yes

Reviewer #2: Yes

3. Has the statistical analysis been performed appropriately and rigorously?

Reviewer #1: I Don't Know

Reviewer #2: Yes

4. Have the authors made all data underlying the findings in their manuscript fully available?

The [PLOS Data policy](https://urldefense.com/v3/__http:/track.editorialmanager.com/CL0/http:*2F*2Fwww.plosone.org*2Fstatic*2Fpolicies.action*23sharing/1/010f019bb6a9522c-a0de53f0-6d1f-47d5-aa6f-fabbf3ad0e8d-000000/lk2j9oJQAYjfNciKeNW2NNVi0rkWXWHd9N_6DJX_zy8=245__;JSUlJSU!!K-Hz7m0Vt54!jozgihvWYEK7lYr3WzzjD1w4SQYsmeLX7hYH3ZKbp7FBgC5P4rulcba8y5rJ3RLADYIF4SoYqnPMF4Y$) requires authors to make all data underlying the findings described in their manuscript fully available without restriction, with rare exception (please refer to the Data Availability Statement in the manuscript PDF file). The data should be provided as part of the manuscript or its supporting information, or deposited to a public repository. For example, in addition to summary statistics, the data points behind means, medians and variance measures should be available. If there are restrictions on publicly sharing data—e.g. participant privacy or use of data from a third party—those must be specified.

Reviewer #1: Yes

Reviewer #2: Yes

5. Is the manuscript presented in an intelligible fashion and written in standard English?

PLOS ONE does not copyedit accepted manuscripts, so the language in submitted articles must be clear, correct, and unambiguous. Any typographical or grammatical errors should be corrected at revision, so please note any specific errors here.

Reviewer #1: Yes

Reviewer #2: Yes

6. Review Comments to the Author

Please use the space provided to explain your answers to the questions above. You may also include additional comments for the author, including concerns about dual publication, research ethics, or publication ethics. (Please upload your review as an attachment if it exceeds 20,000 characters)

Reviewer #1: The authors have provided a very thorough and carefully considered revision, which has substantially improved the manuscript. The simulation studies convincingly underscore the relevance of an estimator that addresses implausible variance differences between trial arms and even provide novel insights in relation to previous methodological work, while also pointing to potentially problematic randomized controlled trials. The discussion of the proposed method is well balanced and appropriately acknowledges its limitations. It would therefore be highly desirable for this method to be considered in applied settings, for example in meta-analyses, to support the assessment of the underlying evidence base and its credibility, ideally via an openly available R package rather than a Git repository alone.

However, as the manuscript has undergone substantial revisions, there are remaining issues related to structure and naming conventions that at times make it difficult to follow.

We thank the reviewer for their comments regarding the substantial improvement of the manuscript and address the remaining feedback below.

In particular, the manuscript appears to pursue four distinct and only loosely connected objectives, each addressed in a separate section:

(1) identification of statistically significant DiVBTA outliers,
(2) simulation studies,
(3) a case study on a landmark fraud case, and
(4) a case study on clinical trials included in systematic reviews on diabetes management.

As a consequence, the manuscript deviates from the conventional IMRaD structure. For example, methods are introduced in both Sections 1 and 2, and the latter simultaneously presents results. Moreover, the two sections appear somewhat disconnected, as estimates or methods introduced in Section 1 do not clearly reappear in Section 2. In this context, closer adherence to established reporting guidelines would substantially strengthen the manuscript. In particular, the recommendations for simulation studies proposed by Boulesteix et al. (1) and Morris et al. (2) (ADEMP framework, endorsed by the STRATOS initiative) would provide a helpful structure.

We thank the reviewer for the suggestion to improve the structure of our manuscript. The manuscript’s structure was fundamentally changed:

1. It now adheres fully the conventional IMRaD structure including the abstract.
2. The ADEMP framework was adhered to in detail and led to fundamental changes in how the methods, results, and supplemental data and figures are presented. Ten separate R-programs were consolidated into one R-program which comprehensively provides all simulations within the framework of ADEMP.

Specifically, we have revised the methods and results, each now focusing on two sections

Methods: (i) Simulation methods within the ADEMP framework and (ii) Case-study assessing real-world utility

Results: (i) Results of the simulation methods within the ADEMP framework and (ii) results of the case-study assessing real-world utility

At present, key elements required for transparent reporting of simulation studies are missing or insufficiently described, including:

- Why are results presented only for LnCVRs?

Three paragraphs are presented in methods on the selection of a statistic quantifying the difference in variance between trial arms (attached below). These three paragraphs provide supportive evidence for viewing lnCVR as a “master” statistic. Other statistics are presented in the supplement (S1 Text).

First, selecting a DiVBTA estimator which can be derived from published summary statistics is crucial given that it remains uncommon for authors of clinical trials to share individual participant data. A 2019 survey of authors from 619 randomized controlled trials published in high-impact anesthesiology journals (2014–2016) found that only about 4% provided individual participant data upon request (28). A 2019 randomized controlled trial assessing the impact of financial incentives to encourage data sharing reported that none of the investigators provided individual participant data (29). As a result, DiVBTA estimators requiring individual participant data for calculations remain currently of little value in identifying potentially problematic trials.

Potential summary statistics of interest to calculate DiVBTA measures are the standard deviation (s), the mean (x$̄$), and the derived measure of coefficient of variation (CV). For a two-arm trial, ${CV}_{T}=\frac{s_{T}}{\bar{\bar{x}_{T}}} \mathrm{and}{CV}_{C}=\frac{s_{C}}{\bar{\bar{x}_{C}}}$ where subscripts T and C denote treatment and control, respectively.

Second, DiVBTA measures which minimize assumption about mean-variance relationships have been recommended over measures which are built on the assumption that no mean-variance relationships exists (30). The log coefficient of variation ratio ($ln\left( \frac{{CV}_{T}}{{CV}_{C}} \right)$ or lnCVR) is from this perspective a conservative choice as it explicitly normalizes variability by the mean. The lnCVR has the other advantage of being a “master” statistic, a statistic which simplifies to other DiVBTA statistics when no mean-variance relationship exists. The log of the variability ratio ($ln\left( \frac{s_{T}}{s_{C}} \right)$ or lnVR) is a special DiVBTA case of lnCVR when group means are equal. The F-ratio ($\frac{s_{T}^{2}}{s_{C}^{2}}$), another DiVBTA ratio measure, is a log transformation of VR (ln(F)=2 lnVR).

And third, normally distributed ratio DiVBTA measures are preferable when it comes identifying outliers. A log transformation can achieve this goal by reducing skewness which is, for instance, inherent to the F-statistic. Ratio DiVBTA measures are preferable to difference DiVBTA measures because they are scale-invariant, robust across heterogeneous studies, and largely unaffected by errors in publications that mislabel standard errors as standard deviations or fail to label the reported measures of variability as either standard errors or standard deviations.

- How was the simulation setup defined with respect to the number of iterations, software packages used, distributional assumptions, and used seeds?

We apologize for the lack of details previously regarding the simulation setup. As indicated above, the ten R-programs included in the prior submission have been consolidated into one R-program providing the necessary information presented in the ADEMP framework. The additional details added in the submitted manuscript is extensive starting on page 10 in the manuscript and ending on page 15.

- How were noise levels and signal-to-noise ratios handled?

We specify effect sizes (the signal) and the noise is generated from the gamma distribution with the parameters derived from a pivotal trial published in JAMA (code to estimate parameters available on GitHub) {Engebretson, 2013 #392}.

- Why was n = 20 chosen for small-sample trials, and is n = 250 a realistic or appropriate choice for large trials?

We selected n=20 for small-sample trials as this was approximately the 25^th^ quantile for sample sizes in trials considered within our diabetes case study (25^th^ quantile for total sample size was 42 which is approximately 20 per trial arm).

| Quantiles (Definition 5) for total sample size |  |
| --- | --- |
| Level | Quantile |
| 100% Max | 11140 |
| 99% | 1486 |
| 95% | 780 |
| 90% | 522 |
| 75% Q3 | 263 |
| 50% Median | 92 |
| 25% Q1 | 42 |
| 10% | 24 |
| 5% | 16 |
| 1% | 10 |
| 0% Min | 5 |

We elected to use n=250 as our large sample size as this was the sample size for a pivotal trial (31). 250 per trial arm is approximately the 90^th^ quantile of sample sizes considered within our diabetes case study.

We apologize for the lack of explanation for this choice and have added this to the Simulation studies section (pp. 13-14); attached below.

Simulations were run in both large-sample (n = 250 per arm) and small-sample (n = 20 per arm) scenarios, reflecting approximate 95th and 25th quantiles of sample sizes in the motivating diabetes trial meta-analysis. For each scenario/combination, 1,000 independent trials (iterations) were simulated which leads to a Monte Carlo standard error for a sensitivity or specificity of 0.95 of ~0.007. The 3-sigma tolerance bounds for outlier classification were derived from a robust meta-analysis of 175 diabetes trials, specifically using the variance of true effects (τ²) and variance of the mean effect (V_M*_) estimated from lnCVR measures in those trials. All simulation code (R), random seeds, and modifiable key parameters are available at <https://www.github.com/mhujoel/DIVBTA> enabling verification and adaptation to other clinical settings.

- Is the chosen range of treatment effect variability (0.2% to 1.4%) plausible for small randomized controlled trials?

We have addressed this important concern by extending the range of plausible treatment effects as, indeed, smaller trials have a wider range of treatment effects. The mean HbA1c improvement (SD) in all trials is -0.34 (SD=0.55). The mean HbA1c improvement (SD) in small trials is -0.49 (SD=0.74). As a result, in response to the reviewer’s point, we have extended the range modeled in this submission from -0.2% to -2.0% which covers the 95% range of improvement for small trials (-0.33 to -2.0).

In addition, clear performance metrics such as sensitivity and specificity are currently missing. Potential users of the proposed method need to understand both the risk of false-positive findings and the probability that an untrustworthy trial remains undetected.

We thank the reviewer for the very important suggestion to add clear performance metrics. As indicated above, we have updated all methods and results to conform to the ADEMP framework and as such have included sensitivity and specificity as performance metrics. These results are now found in Tables 1-3 and additional results in S1 Table, S2 Table, and S3 Table

Minor comments
- Inconsistent naming conventions are used, for example: “Large samples (250/trial arm) and HTE” versus “Small samples (n = 20 per group) and HTE”.

We have revised the manuscript to have consistent naming conventions.

- The correct reference to Tukey’s original work should be included and how fences or spread are defined should be added.

We have now included a reference to the original work by Tukey and changed the terminology in the manuscript and the Figure using the original nomenclature introduced by Tukey. {Tukey, 1977 #481}

Reviewer #2: (No Response)

7. PLOS authors have the option to publish the peer review history of their article ([what does this mean?](https://urldefense.com/v3/__https:/track.editorialmanager.com/CL0/https:*2F*2Fjournals.plos.org*2Fplosone*2Fs*2Feditorial-and-peer-review-process*23loc-peer-review-history/1/010f019bb6a9522c-a0de53f0-6d1f-47d5-aa6f-fabbf3ad0e8d-000000/mf8hy2DxtwgJxpX_U9g6zzjr2Nmwl6D0QShP8TtVqpc=245__;JSUlJSUl!!K-Hz7m0Vt54!jozgihvWYEK7lYr3WzzjD1w4SQYsmeLX7hYH3ZKbp7FBgC5P4rulcba8y5rJ3RLADYIF4SoYUwnKWLA$)). If published, this will include your full peer review and any attached files.

If you choose “no”, your identity will remain anonymous but your review may still be made public.

**Do you want your identity to be public for this peer review?** For information about this choice, including consent withdrawal, please see our [Privacy Policy](https://urldefense.com/v3/__https:/track.editorialmanager.com/CL0/https:*2F*2Fwww.plos.org*2Fprivacy-policy/1/010f019bb6a9522c-a0de53f0-6d1f-47d5-aa6f-fabbf3ad0e8d-000000/7IR7Q9bOBjbWyylzd2QN2UMfnewzXmmUf8rbQ9QaLNI=245__;JSUl!!K-Hz7m0Vt54!jozgihvWYEK7lYr3WzzjD1w4SQYsmeLX7hYH3ZKbp7FBgC5P4rulcba8y5rJ3RLADYIF4SoYxyDyhKQ$).

Reviewer #1: **Yes:** Dr. Adrian Richter

Reviewer #2: **Yes:** Emma Sydenham
